# Comprehensive Assessment of the KDM2B‐Associated Neurodevelopmental Disorder and the 12q24.31 Microdeletion Syndrome

**DOI:** 10.1111/cge.70169

**Published:** 2026-04-09

**Authors:** Amber S. E. van Oirsouw, Tzung‐Chien Hsieh, Martijn Koetsier, Abdulrazak Alali, Fatimah Albuainain, Elena Bacchelli, Tahsin Stefan Barakat, Yline Capri, Sandra Chantot‐Bastaraud, Valeria Capra, Deanna Alexis Carere, Emma Clement, Nour Elkhateeb, Madeleine Franchi, Jing‐Mei Li, Nicole Matthews, Vanda McNiven, Sarju G. Mehta, Masayuki Nakamura, Chanika Phornphutkul, Nicole Revencu, Marcello Scala, Natalie Shallow, Jennifer Stefanich, Marta Viggiano, Paola Visconti, Susan Walker, Federico Zara, Mariëlle Alders, Bobby P. C. Koeleman, Renske Oegema

**Affiliations:** ^1^ Department of Genetics University Medical Center Utrecht, Utrecht University Utrecht the Netherlands; ^2^ Institute for Genomic Statistics and Bioinformatics, University Hospital Bonn, Rheinische Friedrich‐Wilhelms‐University Bonn Bonn Germany; ^3^ West Virginia University Morgantown West Virginia USA; ^4^ Department of Clinical Genetics Erasmus MC University Medical Center Rotterdam the Netherlands; ^5^ Department of Pharmacy and Biotechnology University of Bologna Bologna Italy; ^6^ IRCCS Istituto delle Scienze Neurologiche di Bologna Bologna Italy; ^7^ Department of Genetics APHP Nord‐University Hospital Robert Debré Paris France; ^8^ Department of Génétics APHP‐Hopital La Pitié Salpétrière Paris France; ^9^ Genomics and Clinical Genetics Unit, IRCCS Istituto Giannina Gaslini Genoa Italy; ^10^ GeneDx, LLC Gaithersburg Maryland USA; ^11^ Department of Clinical Genetics Great Ormond Street Hospital for Children London UK; ^12^ Department of Clinical Genetics Cambridge University Hospitals NHS Foundation Trust Cambridge UK; ^13^ UF Health Jacksonville Genetics, University of Florida Jacksonville Florida USA; ^14^ Division of Genetics, Department of Pediatrics McMaster University Hamilton Canada; ^15^ Genetics Program, Peterborough Regional Health Centre Peterborough Canada; ^16^ Department of Psychiatry Graduate School of Medical and Dental Sciences, Kagoshima University Kagoshima Japan; ^17^ Division of Human Genetics, Rhode Island Hospital/Hasbro Children's, Brown University Providence Rhode Island USA; ^18^ Center for Human Genetics, Cliniques universitaires Saint‐Luc, Université catholique de Louvain Brussels Belgium; ^19^ Department of Neurosciences, Rehabilitation, Ophthalmology, Genetics, Maternal and Child Health Università di Genova Genoa Italy; ^20^ Medical Genetics Unit, IRCSS Istituto Giannina Gaslini Genoa Italy; ^21^ Genetic Center, Akron Children's Hospital Akron Ohio USA; ^22^ IRCCS Istituto delle Scienze Neurologiche di Bologna, UOSI Disturbi dello Spettro Autistico Bologna Italy; ^23^ Genomics England London UK; ^24^ Department of Human Genetics Amsterdam UMC, University of Amsterdam Amsterdam the Netherlands; ^25^ Reproduction and Development Research Institute Amsterdam the Netherlands

**Keywords:** 12q24.31 microdeletion, EpiSign, KDM2B, neurodevelopmental disorder

## Abstract

The recently delineated KDM2B‐associated neurodevelopmental disorder (NDD) is characterized by developmental delay and variable co‐morbidity. Genotype–phenotype correlations are emerging, in particular a distinct clinical presentation caused by CxxC domain variants. We report here a novel intragenic deletion which leads to in vitro expression of a shortened KDM2B protein lacking the complete CxxC domain. In addition, we present data on 12 other individuals; two with larger 12q24.31 microdeletions, one with a frameshift variant, and nine with missense variants. We analyzed genotype–phenotype correlations of this cohort combined with previously reported individuals (*n* = 68) and classify 37 variants in 47 individuals as pathogenic or likely pathogenic. We observe a highly penetrant CxxC‐related phenotype with distinct facial features supported by GestaltMatcher. In contrast, our findings point to variable expressivity and incomplete penetrance of loss‐of‐function variants and JmjC domain variants complicating variant classification and genetic counseling. We identify *KDM2B* as a strong contributor to the 12q24.31 microdeletion syndrome, while also addressing the role of additional genes in the region. Thus, our study defines the KDM2B‐NDD's clinical spectrum and highlights the importance of integrating molecular, (epi)genetic, and phenotypic data in NDD diagnostics.

## Graphical abstract

We set out to characterize genotype–phenotype correlations in the recently delineated KDM2B‐associated neurodevelopmental disorder. We observe a highly penetrant CxxC domain‐related phenotype with distinct facial features supported by GestaltMatcher. In contrast, our findings point to variable expressivity and incomplete penetrance of loss‐of‐function variants and JmjC domain variants complicating variant classification and genetic counseling. Also, we identify *KDM2B* as a strong contributor to the 12q24.31 microdeletion syndrome.

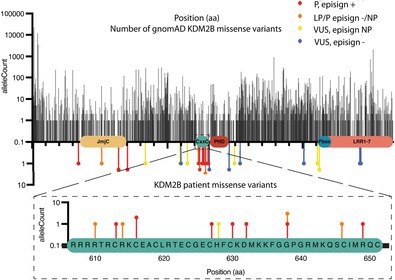

**Abbreviations**
ADHD: attention deficit hyperactivity disorder
ASD: autism spectrum disorder
DD: developmental delay
F: female
GFP: green fluorescent protein
ID: intellectual disability
LP: ACMG likely pathogenic
LRR: leucine‐rich region
M: male
MODY3: Maturity Onsent of Diabetes of the Young Type 3
N: number of individuals
NA: not assessed
NC: negative control
NDD: neurodevelopmental disorder
NMD: nonsense‐mediated decay
OFC: occipital frontal circumference
P: ACMG pathogenic
PDA: patent ductus arteriosus
PFO: patent foramen ovale
pLOF: putative loss‐of‐function
PTSD: post‐traumatic stress disorder
SD: standard deviation
UK: unknown
VSD: ventricular septal defect
VUS: ACMG variant of uncertain significance
VUS: variant of uncertain significance
WT: wild‐type

## 1 Introduction

Variants in *KDM2B*, encoding the epigenetic regulator lysine‐demethylase 2B (KDM2B; MIM *609078) cause a recently delineated NDD [1]. The canonical KDM2B isoform (KDM2B‐LF) contains a JmjC domain capable of demethylating histone lysines K4, K36, and K79 [2, 3, 4], a CxxC domain that binds unmethylated CpG dinucleotides [5, 6], and an F‐box domain and leucine‐rich repeat (LRR) region involved in protein–protein interactions [7, 8]. Multiple alternative isoforms exist [9], including a short isoform lacking the JmjC domain, which is expressed during early murine development [7, 10].

Previous reports have described heterozygous loss‐of‐function (LOF) and missense variants in *KDM2B* in individuals presenting with a NDD [1, 11, 12, 13, 14]. Notably, variants affecting the CxxC domain are associated with a distinct NDD with congenital cardiac defects and variable renal and ocular abnormalities (OMIM # 621474) [14].

*KDM2B* is included with the 12q24.31 microdeletion syndrome region. Currently, 13 individuals have been described with 12q24.31 microdeletions ranging from 360 kb to 1.7 Mb and a broadly overlapping phenotype of DD, ID, ASD, anxiety, epilepsy, and craniofacial anomalies [15, 16, 17, 18, 19, 20, 21, 22, 23]. Based on the smallest deletion, the epigenetic regulators *KDM2B* and *SETD1B* have been proposed as candidate genes responsible for the phenotype [15]. Pathogenic variants in *SETD1B* have been associated with an NDD (MIM 619000) [24]. For both *KDM2B*‐ and *SETD1B*‐related syndromes an episignature has been identified [1, 16].

In this study, we report 13 previously unpublished individuals and review literature to compile a comprehensive overview of individuals with *KDM2B* variants. We use GestaltMatcher to analyse facial features and test selected samples for the *KDM2B*‐specific episignature. We analyse the genotype–phenotype correlations of the *KDM2B*‐associated disorder and compare these results to the clinical and genetic data of the 12q24.31 microdeletion syndrome.

## 2 Materials and Methods

### 2.1 Patient Inclusion and Data Collection

This study is approved by the medical ethical committee of the University Medical Centre Utrecht (TCBIO 21‐355, March 18, 2021). Inclusion required the presence of a KDM2B variant or a ≤ 2 Mb 12q24.31 microdeletion. The *KDM2B* cohort was assembled through international collaborations, the Genomics England cohort [25], the GeneMatcher platform [26], the European Reference Network for Intellectual disability, TeleHealth, Autism and Congenital Anomalies (ERN‐ITHACA) and a PubMed literature search [1, 11, 12, 13, 14, 15, 16, 17, 18, 19, 20, 21, 22, 23, 27]. Informed consent for publication was obtained from all participants or their legal representatives.

### 2.2 Analysis of *KDM2B* Variants

Population variant frequencies and gene constraint scores were obtained from gnomAD v4.1.0 [28] and RGC v1.1.2 [29]. *In silico* prediction of variant impact was performed using Polymorphism Phenotyping v2 (PolyPhen‐2) [30], Sorting Intolerant from Tolerant (SIFT) [31], AlphaMissense [32], MutationTaster [33], and MetaDome [34]. Variant classification followed the 2015 American College of Medical Genetics and Genomics (ACMG) and the Association for Molecular Pathology guidelines [35]. The criteria application and variant classification presented in this paper does not necessarily reflect the criteria and classification of the clinical testing laboratories involved in this paper. Whole blood‐derived DNA samples were collected from study participants. Methylation data were analyzed using the EpiSign assay, as described previously [1, 36]. Processed beta values were put into a support vector machine (SVM) classifier that compares the patient methylation profile with methylation profiles from reference disorder‐specific cohorts and unaffected controls. The final result is determined based on three evaluated parameters: MVP scores, hierarchical clustering, and multidimensional scaling (MDS). A result is considered to have high confidence positive when there is concordance across all three parameters. In addition, the presence of *SETD1B*‐associated episignature was assessed for the 12q24.31 microdeletion cases [16].

### 2.3 Pathogenicity Region Analysis

Analysis of *KDM2B* regions associated with pathogenicity was performed using a combination of resources: MetaDome Tolerance Landscape [34], AlphaMissense pathogenicity scores [32], population variant frequencies from gnomAD v4.1.0 [28], *KDM2B* cohort genetic data, and EpiSign results.

### 2.4 GestaltMatcher Facial Analysis

We collected facial photographs of 17 individuals. Facial photographs were analyzed using GestaltMatcher [37, 38]. Methods are described in the Supporting Information.

### 2.5 DNA Constructs and Protein Expression in HEK293T Cells

The DNA constructs and methods for expression analysis were performed as previously described [14] (Supporting Information Methods).

## 3 Results

### 3.1 KDM2B Episignature

We performed EpiSign testing in 9 individuals (6 novel and 3 previously published cases).

We listed all current and previous EpiSign results in Table S3. In total, 23 variants tested positive. Conversely, eight missense variants and one putative splice variant tested negative.

### 3.2 Intragenic Deletion

**FIGURE 1:**
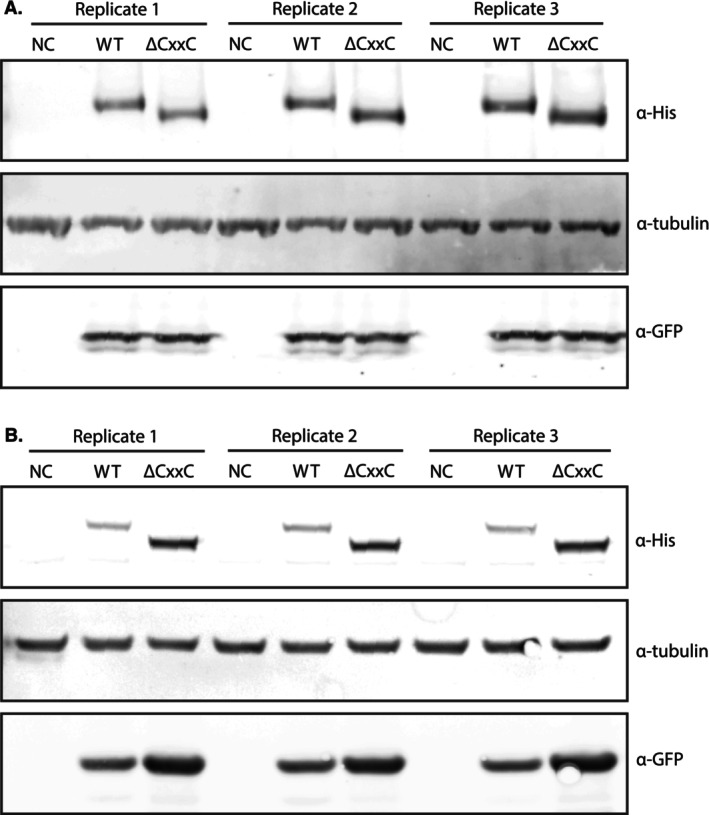
Expression of KDM2B long (LF) and short form (SF) with the CxxC deletion (ΔCxxC). (A, B) Western blot results of wild‐type (WT) and variant His‐KDM2B‐LFΔCxxC (A) and variant His‐KDM2B‐SFΔCxxC (B) expression in HEK293T cells following transfection with WT or variant His‐KDM2B expression plasmid (*n* = 3 replicates). Tubulin was used as a loading control, GFP as transfection control. An untransfected sample was also included (NC). Abbreviations: GFP, green fluorescent protein; NC, negative control; WT, wild‐type.

The p.(Asn579_Ala653del) deletion in individual #3 is predicted to cause an in‐frame deletion of the CxxC domain. We overexpressed wild‐type and mutant short (SF) and long form (LF) *KDM2B* constructs in HEK293T cells. A smaller mutant protein was observed, with a size difference consistent with the expected 8.3 kDa loss of its CxxC domain. This mutant protein showed comparable expression levels to wild‐type (Figure 1), both in the absence and presence of a proteasome inhibitor (Figure S1). These findings demonstrate that KDM2B lacking its DNA‐binding CxxC domain can be expressed in vitro and suggest that it is not prone to premature degradation.

### 3.3 Novel Patients

**FIGURE 2:**
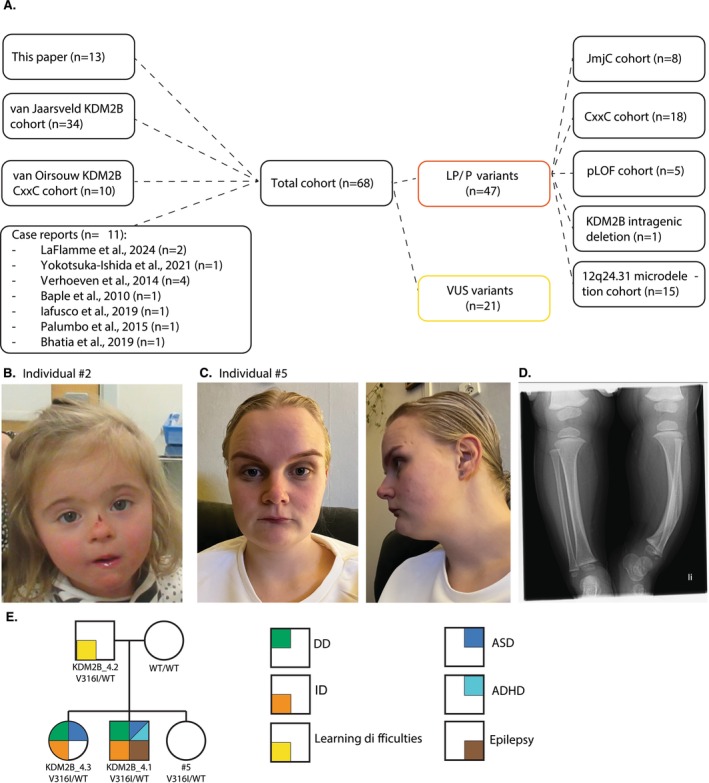
A cohort of individuals with variants affecting *KDM2B*. (A) Flow chart of all individuals included in this study (*n* = 68). The van Jaarsveld KDM2B cohort includes previously published individuals [11, 12, 13, 15, 16, 22, 23]. (B) Facial photograph of individual #2. (C) Facial photographs of individual #5. (D) X‐ray of the lower extremities of individual #5 demonstrating unilateral bowing of the tibia. (E) Pedigree of the family of individual #5. Abbreviations: ADHD, attention deficit hyperactivity disorder; ASD, autism spectrum disorder; DD, developmental delay; ID, intellectual disability; pLOF, putative loss‐of‐function; WT, wild‐type; VUS, ACMG variant of uncertain significance; LP, ACMG likely pathogenic; P, ACMG pathogenic.

We identified 13 previously unreported individuals of whom 11 had a novel variant (Table S1) and classified all novel variants according to the ACMG criteria [35] (Figure 2B–D, Tables S1, S4). Six novel individuals (#1–6) have a (likely) pathogenic variant; the p.(Asn579_Ala653del) deletion described above, two 12q24.31 microdeletions, one frameshift variant, and three missense variants (Clinical reports presented in Supplements). Individual #5 is from a previously reported family (Figure 2E) [1]. Seven individuals have a variant of unknown significance (VUS) (#7–13).

### 3.4 KDM2B's Variant Spectrum

We analyzed KDM2B's variant spectrum and included all novel and reported variants. In 68 individuals, a total of 53 variants were identified, 37 of which were classified as pathogenic or likely pathogenic (Figure 2A, Table S2).

**FIGURE 3:**
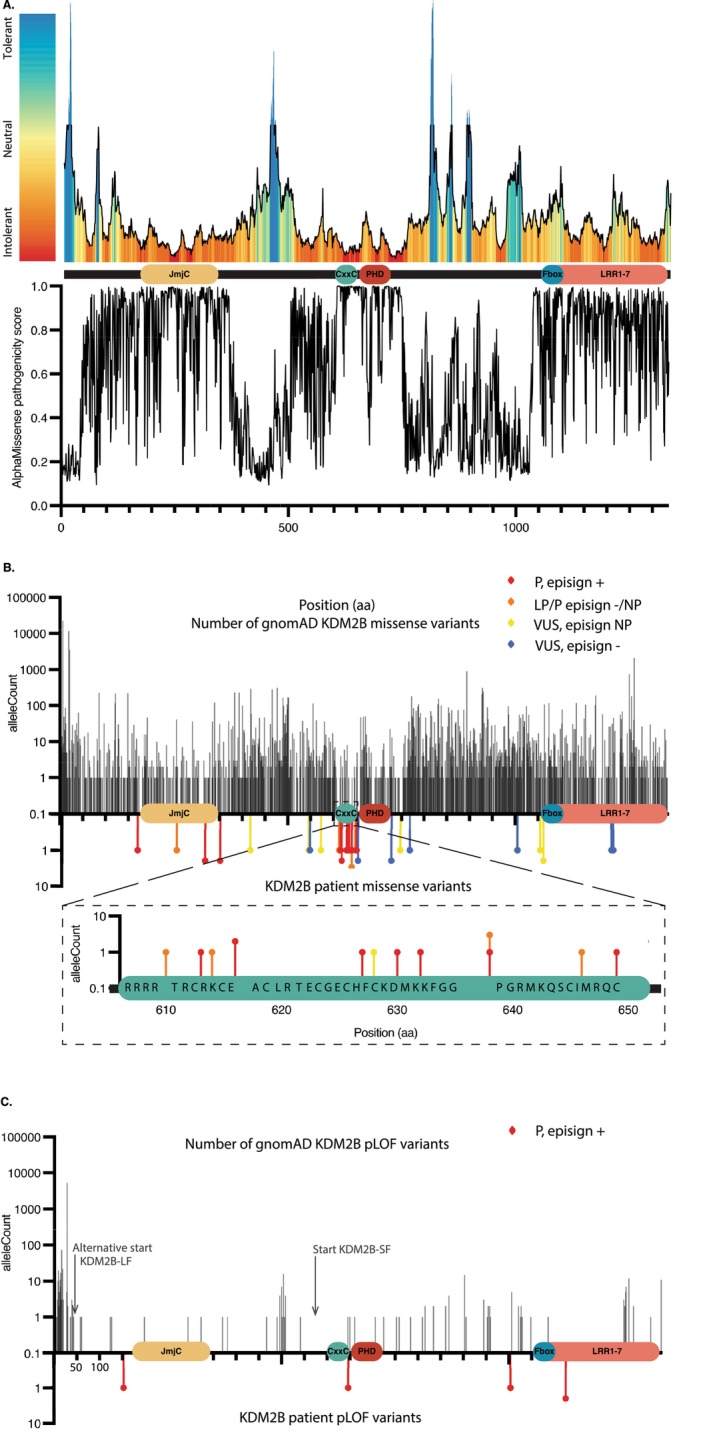
Distribution of population and patient variants in *KDM2B*. (A) MetaDome tolerance landscape of KDM2B [34] and AlphaMissense pathogenicity scores [32] along a linear protein representation showing its functional domains (UniProt Q8NHM5), demonstrating regions of reduced tolerance and increased pathogenicity scores. AlphaMissense pathogenicity scores represent the average pathogenicity score per amino acid and are plotted by protein position. (B, C) Distribution of missense variants (B) and pLOF variants (C) from gnomAD v4.1.0 [28] compared with pathogenic variants identified in affected individuals along the linear protein representation of KDM2B (UniProt Q8NHM5). alleleCount (*y*‐axis) is the sum of gnomAD (upper part graph) or patient (lower part graph) missense or pLOF variants by amino acid position. The CxxC domain is enlarged to amino acid position in (B); the alternative start of KDM2B‐LF and start of KDM2B‐SF is indicated in (C). KDM2B cohort variants are color‐coded based on EpiSign results and ACMG classification. Abbreviations: aa, amino acid; LF, long form; LRR, leucine‐rich region; NP = not performed; pLOF, putative loss‐of‐function; SF, short form; VUS, ACMG variant of uncertain significance; LP, ACMG likely pathogenic; P, ACMG pathogenic.

We identified clustering of pathogenic missense variants (ACMG class 4 or 5) within or in proximity to the JmjC and CxxC domains. These domains show the highest intolerance to variation [34] and are strongly associated with pathogenicity by AlphaMissense [32] (Figure 3A). In contrast, VUSes were identified outside these domains, of which 7 of 7 tested negative on EpiSign. Population missense variation from gnomAD v4.1.0 is reduced in the JmjC domain (464/57655; 0.80%, despite comprising 12.57% of KDM2B) and the CxxC domain (34/57655; 0.06%, despite comprising 3.44% of KDM2B, Figure 3B).

*KDM2B* is intolerant of pLOF variants (pLi = 1, o/e = 0.19 (0.14–0.27)) in the general population [28]. The vast majority (5511/5657; 97.4%) of pLOF are located in exon 1 (Figure 3C), where nonsense‐mediated decay (NMD) is inefficient [39]. Moreover, expression data [9] indicate the presence of an alternative start codon between exons 1 and 2 (arrow in Figure 3C). Additional pLOF clusters are observed in the final (13/5657; 0.2%) and penultimate exon (31/5657; 0.5%), with low predicted efficiency of NMD [39], and around position 500 (34/5657, 0.6%) in exon 11 of the canonical transcript (ENST00000377071.9 = NM_032590.5), upstream of the short form KDM2B‐SF and downstream of short JmjC isoforms.

### 3.5 12q24.31 Microdeletions

**FIGURE 4:**
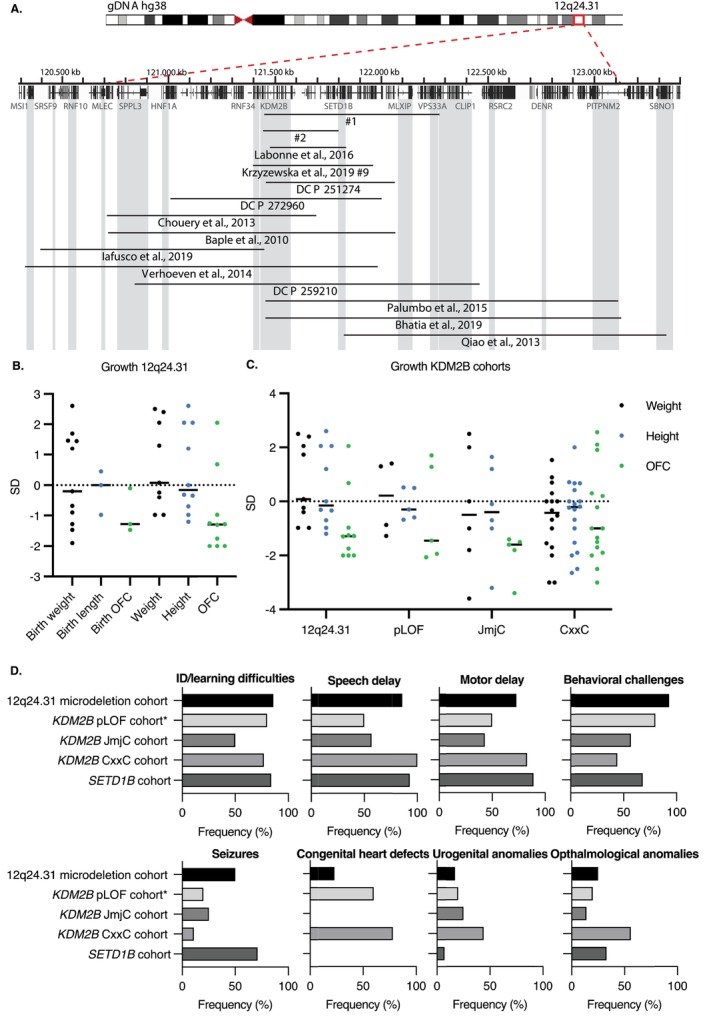
Cohort analysis of KDM2B‐associated disorders. (A) Genomic locations of 12q24.31 microdeletions mapped onto a linear representation. In light grey, the location of haploinsufficient genes is indicated (loss‐of‐function observed/expected upper bound fraction (LOEUF) threshold of < 0.6). (B) Growth measurements at birth or at last investigation of individuals with 12q24.31 microdeletions, expressed in standard deviations (SD), reveal a trend towards below‐average OFC. (C) Growth measurements at last investigation of 12q24.31 microdeletion, pLOF, JmjC and CxxC cohorts, expressed in standard deviations (SD). (D) Summary of clinical features across 12q24.31 microdeletion, pLOF, JmjC, CxxC and SETD1B cohorts, presented as bar graphs indicating the frequency of the main phenotypic findings. The asterisk * indicates to interpret the frequency of *KDM2B* pLOF cohort with caution, as two individuals with additional diagnosis are included (Noonan syndrome and Phelan‐McDermid syndrome). Abbreviations: OFC, occipital frontal circumference; pLOF, putative loss‐of‐function.

We analyzed the data from individuals #1, #2, and 13 published cases with 12q24.31 microdeletions [15, 16, 17, 18, 19, 20, 21, 22, 23]. The deletion size ranged from 356 kb to 1.66 Mb, without a common overlapping region (Figure 4A). *De novo* occurrence was confirmed in 10 of 15 cases, while one deletion was inherited from an unaffected father [23]. One deletion did not encompass KDM2B and was included in the analysis because of a positive *KDM2B* episignature potentially resulting from disruption of *KDM2B* regulatory regions [1].

The core phenotype of the 12q24.31 microdeletion syndrome includes DD, ID, behavioral problems, and various dysmorphisms (Tables 1, 2; Table S5, Supporting Information Text).

**TABLE 1:** Clinical data of 15 individuals with 12q24.31 microdeletions.

	#1	#2	Baple	Bhatia	Chouery	Chouery (father)	Iafusco	Krzyzewska #9	Labonne	Palumbo	Qiao #2	Verhoeven	Verhoeven/Labonne #DCP 251274	Verhoeven/Labonne #DCP 259210	Verhoeven/Labonne #DCP 272960
Sex, age	F, 9 years	F, 3 years 2 months	F, 13 years	M, 4 years	F, 12 years 6 months	M, adult	F, 10 years	F, 13 years	M, 9 years 7 months	F, 11y	F, 14 years 10 months	M, 39 years	F, 13 years	F, 14 years	F, 7years
GDD	+	+	+	+	+	−	+	+	+	+	+	+	+	+	+
ID	+	+	+	NA	+	−	+	+	+	+	+	−	+	+	+
Motor delay	+	+	−	NA	+	−	+	—	+	+	+	+	NA	NA	NA
Speech/language delay	+	+	+	NA	+	−	+	+	+	+	+	+	+	+	−
Behavioral problems	ASD, ADHD	Autistic features	ASD, anxieties	Anxiety	Social interaction skills lacking	−	ASD, anxiety	ASD, ADHD	ASD, anxiety	Stereotypies, anxieties	ASD, selective mutism, anxiety	Social function suboptimal	ASD	ASD	NA
Cardiovascular	−	−	−	Large atrial septal defect, tiny PDA	−	−	Congenital interatrial defect	−	−	PFO, mild mitral regurgitation	Heart size at upper limit of normal	−	NA	NA	NA
Other	−	Macroglossia, cutis marmorata	Macroglossia	Macroglossia	Macroglossia, Bilateral hip luxation	Insulin dependent diabetes	Macroglossia, hyperlordosis, Hashimoto's thy‐roiditis, scoliosis	Macroglossia	−	Macroglossia	Pneumonia, T‐cell skin lymphoma	Cryptorchidism, PKD, delayed bone age	Cutis marmorata	NA	NA
Ophthalmalogical	−	Right eye esotropia. Refractive error.	−	Myopia	−	−	−	−	−	−	Strabismus	Mild diabetic non‐proliferative retinopathy	NA	NA	NA
Dysmorphism	+	+	+	+	+	−	+	+	+	+	+	+	+	−	NA
Hypotonia	−	+	NA	+	+	−	+	−	+	−	+	NA	−	−	NA
Seizures	+	−	−	−	+	−	+	NA	+	+	−	−	+	−	+

Abbreviations: ADHD, attention deficit hyperactivity disorder; ASD, autism spectrum disorder; F, female; GDD, global developmental delay; ID, intellectual disability; M, male; NA, not assessed; PDA, patent ductus arteriosus; PFO, patent foramen ovale.

**TABLE 2:** Comparison of clinical features according to genotype.

FEATURES Frequency (%)	12q24.31 microdeletion cohort	*KDM2B* pLOF cohort^a^	*KDM2B* JmjC cohort	*KDM2B* CxxC cohort	*SETD1B* cohort [24]
	*n* = 15	*n* = 5	*n* = 8	*n* = 18	*n* = 28
Development					
ID (HP:0001249)/learning difficulties	12/14 (86%)	4/5 (80%)	4/8 (50%)	10/13 (77%)	21/25 (84%)
Motor delay (HP:0001270)	8/11 (73%)	2/4 (50%)	3/7 (43%)	15/18 (83%)	25/28 (89%)
Speech delay (HP:0000750)	12/14 (86%)	2/4 (50%)	4/7 (57%)	18/18 (100%)	26/28 (93%)
Behavioral challenges	13/14 (93%)	4/5 (80%)	4/7 (57%)	8/18 (44%)	19/28 (68%)
ASD (HP:0000729)/autistic features	10/14 (71%)	3/5 (60%)	2/7 (29%)	6/18 (33%)	18/28 (64%)
Anxiety (HP:0000739)	6/14 (43%)	0/5 (0%)	1/7 (14%)	1/16 (6%)	7/27 (26%)
ADHD (HP:0007018)	2/14 (14%)	1/5 (20%)	2/7 (29%)	3/16 (19%)	9/27 (33%)
Seizures (HP:0001250)	7/14 (50%)	1/5 (20%)	2/8 (25%)	2/18 (11%)	20/28 (71%)
Growth					
Hypoglycemia	2/14 (14%)	UK	UK	2/18 (11%)	7/26 (27%)
Microcephaly (HP:0000252)	3/11 (27%)	1/5 (20%)	1/8 (13%)	4/15 (27%)	1/27 (4%)
Macrocephaly (HP:0000256)	1/11 (9%)	0/5 (0%)	1/8 (13%)	2/15 (13%)	2/27 (7%)
Organ system anomalies					
Congenital heart defects	3/13 (23%)	3/5 (60%)	0/8 (0%)	14/18 (78%)	0/28 (0%)
Atrial septal defect (HP:0001631)	3/13 (23%)	0/5 (0%)	0/8 (0%)	11/18 (61%)	0/28 (0%)
PFO (HP:0001655)	1/13 (8%)	0/5 (0%)	0/8 (0%)	5/18 (28%)	0/28 (0%)
VSD (HP:0001629)	0/13 (0%)	2/5 (40%)	0/8 (0%)	4/18 (22%)	0/28 (0%)
Pulmonary stenosis (HP:0034350)	0/13 (0%)	0/5 (0%)	0/8 (0%)	4/18 (22%)	0/28 (0%)
PDA (HP:0001643)	1/13 (8%)	0/5 (0%)	0/8 (0%)	4/18 (22%)	0/28 (0%)
Urogenital anomalies	2/12 (17%)	1/5 (20%)	2/8 (25%)	8/18 (44%)	2/28 (7%)
Single kidney (HP:0000122)	0/12 (0%)	0/5 (0%)	0/8 (0%)	5/18 (28%)	0/28 (0%)
Opthalmological anomalies	3/12 (25%)	1/5 (20%)	1/7 (14%)	10/18 (56%)	9/27 (33%)
Myopia (HP:0000545)	1/12 (8%)	0/5 (0%)	0/7 (0%)	4/18 (22%)	2/27 (7%)
Nasolacrimal duct stenosis (HP:0007678)	0/12 (0%)	0/5 (0%)	0/7 (0%)	3/18 (17%)	0/27 (0%)
Strabismus	1/12 (8%)	0/5 (0%)	1/7 (14%)	2/18 (11%)	4/27 (15%)
Astigmatism	0/12 (0%)	1/5 (20%)	0/7 (0%)	2/18 (11%)	3/27 (11%)
Amblyopia	0/12 (0%)	0/5 (0%)	0/7 (0%)	0/18 (0%)	2/27 (7%)
Abnormal brain imaging	2/5 (40%)	2/4 (50%)	1/3 (33%)	5/10 (50%)	5/23 (22%)
Cerebral cysts	0/5 (0%)	0/4 (0%)	0/3 (0%)	3/10 (30%)	UK
Ventriculomegaly (HP:0002119)	0/5 (0%)	0/4 (0%)	0/3 (0%)	3/10 (30%)	UK
Other					
Facial dysmorphism	12/14 (86%)	4/5 (80%)	5/7 (71%)	17/18 (94%)	23/27 (85%)
Macroglossia (HP:0000158)/protruding tongue	8/13 (62%)	0/5 (0%)	0/7 (0%)	1/18 (6%)	1/27 (4%)
Skeletal and limbs	2/12 (17%)	0/5 (0%)	3/7 (43%)	12/18 (67%)	13/28 (46%)
Pes planovalgus (HP:0001763)	0/12 (0%)	0/5 (0%)	0/6 (0%)	5/18 (28%)	2/28 (7%)
Hypotonia (HP:0001252)	6/12 (50%)	2/3 (66%)	0/2 (0%)	7/16 (44%)	14/27 (52%)
Feeding difficulties (HP:0011968)	3/3 (100%)	UK	1/4 (25%)	6/11 (55%)	2/28 (7%)

Abbreviations: ADHD, attention deficit hyperactivity disorder; ASD, autism spectrum disorder; ID, intellectual disability; PDA, patent ductus arteriosus; PFO, patent foramen ovale; UK, unknown; VSD, ventricular septal defect.

^a^ In the pLOF cohort two individuals with additional diagnosis are included (Noonan syndrome and Phelan‐McDermid syndrome).

### 3.6 12q24.31 Candidate Gene Analysis

The deletions spanned a total of 72 protein‐ and non‐protein‐coding genes of which 16 have a loss‐of‐function observed/expected upper bound fraction (LOEUF) threshold of < 0. (Table S6). This includes six with a link to human disorders: *KDM2B*‐ and *SETD1B*‐associated monogenic NDD, *HNF1A* implicated in autosomal dominant maturity‐onset diabetes of the young Type 3 (MODY3), and *VPS33A* linked to autosomal recessive mucopolysaccharidosis‐plus syndrome. Heterozygous missense variants in *DENR* and homozygous nonsense variants in *CLIP1* have been identified in individuals with ID/NDD (Table S6), though definitive evidence for their causative role remains lacking [40, 41, 42, 43, 44].

### 3.7 Genotype–Phenotype Correlations

We grouped individuals based on KDM2B genotype: 12q24.31 microdeletions, pLOF variants, JmjC variants, and CxxC variants and compared these with 28 previously described individuals with pathogenic *SETD1B* variants [24].

All cohorts shared core features of DD with ID and facial dysmorphism (Table 2, Figure 4B–D). Behavioral challenges were present in most individuals except for those individuals with CxxC variants (8/18). Epilepsy is present in half of the individuals with 12q24.31 microdeletions (7/14), as compared with a majority in the *SETD1B* cohort (20/28) and a minority in the *KDM2B* cohorts (1/5 pLOF, 2/8 JmjC, 2/18 CxxC). Growth data showed a tendency towards a small head circumference (Figure 4C). Congenital heart defects were present in 12q24.31 (3/12) and *KDM2B* (14/18 CxxC, 3/5 pLOF) cohorts, while they were absent in the *KDM2B* JmjC and *SETD1B* cohorts.

This cohort analysis supports genotype–phenotype correlations in *KDM2B*‐associated syndromes. JmjC and pLOF variants showed incomplete penetrance and variable expressivity, with DD/ID present in about half of the individuals. In contrast, CxxC variants were associated with a more consistent and extensive phenotype. The findings also support *KDM2B* as an important contributor to the 12q24.31 microdeletion phenotype. All reported deletions affected *KDM2B*, and individuals without deletions involving *SETD1B* or other shared haploinsufficient genes still presented with features such as DD/ID, behavioral challenges, facial dysmorphism, and epilepsy. However, the presence of additional features—such as macroglossia, which is largely absent in both *KDM2B* and *SETD1B* cohorts—suggests that neither gene is likely to be the sole or primary driver. This implicates the involvement of other genes within the deleted region in shaping the full phenotypic spectrum.

Finally, we compared the phenotype of individual #3 with the in‐frame deletion of the CxxC domain to the different KDM2B‐associated subgroups. This individual presented with global DD, ID, ASD, anxiety, pes planovalgus, and congenital anomalies affecting the heart, urogenital tract and eyes. He also had downslanted palpebral fissures, midface retrusion, and large ear lobes. These clinical features are highly consistent with those of the *KDM2B* CxxC cohort, further supporting a shared molecular impact—that is, loss of DNA‐binding ability—of CxxC missense variants and CxxC domain loss [14].

### 3.8 GestaltMatcher Facial Analysis

The GestaltMatcher dataset consisted of seven individuals with CxxC variants, four with JmjC variants, one with a pLOF variant, and six with 12q24.31 microdeletions. The individual with a pLOF variant did not cluster with individuals from other groups (data not shown) and was excluded from further analysis.

**FIGURE 5:**
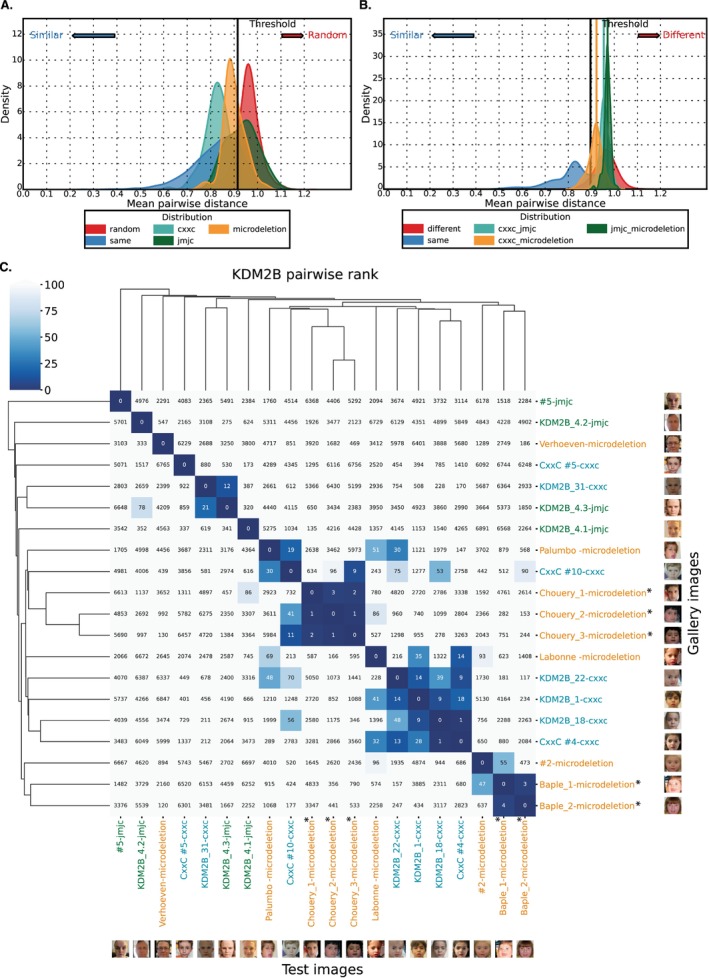
GestaltMatcher analysis. (A) Intra‐cohort mean pairwise cosine distances among individuals with KDM2B‐related variants. Each distribution shows the facial similarity within the groups Lower values indicate higher similarity. (B) Distribution of inter‐cohort cosine distances between groups. Each distribution represents pairwise cosine distances between two while most inter‐cohort comparisons show high distances (i.e., low similarity), a subset of CxxC–microdeletion pairs shows low distances (left tail), suggesting partial facial similarity (C) Pairwise rank matrix showing facial similarity. Each cell shows the rank of similarity based on cosine distance—lower values indicate higher facial similarity, with rank 1 being the closest match. The matrix is interpreted column‐wise. CxxC individuals show strong mutual similarity. * multiple facial photographs of this individual are included.

Intra‐cohort analysis (Figure 5A) showed that the CxxC group exhibited the most consistent facial gestalt, with 97.5% of pairwise distances falling below the similarity threshold (c = 0.915). The microdeletion group also showed moderate consistency (70.2% below threshold), whereas only 36.4% of JmjC pairwise comparisons fell below the threshold.

Inter‐cohort comparisons (Figure 5B) % of JmjC–microdeletion, and 90% of CxxC–microdeletion pairwise distances were above the threshold. However, select CxxC–microdeletion pairs showed lower distances. The pairwise rank matrix (Figure 5C) confirmed these trends: CxxC individuals clustered tightly, with two ranking 1st for each other. In summary, these findings point to a *KDM2B* CxxC distinct and consistent facial gestalt.

## 4 Discussion

In this study, we characterized the clinical and genomic spectrum of the KDM2B‐associated disorder by analyzing data of 47 individuals with 37 pathogenic or likely pathogenic variants. The spectrum divides into 4 major groups: missense variants in the JmjC and CxxC domains, pLOF variants, and 12q24.31 microdeletions. Notably, we report the first individuals with bilateral iridocorneal adhesions, tibial bowing, craniosynostosis and splenic cysts. Currently, evidence is lacking for the pathogenicity of missense variants outside the JmjC and CxxC domains. None of these variants have been functionally assessed and 7/7 tested negative for the KDM2B episignature. Further studies are needed to determine whether these variants are benign or cause disease through an alternate mechanism not associated with the *KDM2B* episignature.

The GestaltMatcher results underscore our previous observation of similar facial characteristics in the CxxC cohort, consisting of downslanted palpebral fissures, a bulbous nasal tip, a prominent cupid's bow, full cheeks, asymmetric or dysplastic ears and prominent ear lobes [14]. Representative images are shown in Figure S2. Of note, the GestaltMatcher analysis remains largely similarity‐based and does not identify the specific facial traits underlying the observed clustering. For the JmjC and microdeletion groups we observed marked heterogeneity. The results should be interpreted cautiously due to small subgroup sizes within variant groups. A re‐evaluation in the future, including more patients, is required. Future work using explainable AI approaches to connect computational similarity to feature‐level dysmorphology (e.g., mapping salient regions to standardized phenotypic terms) would enhance interpretability and clinical usefulness to understand what facial features are really shared among the patients.

For JmjC and pLOF variants we observed incomplete penetrance. Although most variants occurred *de novo*, all four inherited variants were either pLOF or located in the JmjC domain [1, 23, 27]. These variants were all absent or present at low frequency in the population, were predicted to be damaging by *in silico* tools, and were all associated with a positive KDM2B episignature.

Recent literature increasingly recognizes incomplete penetrance and variable expressivity in monogenic NDD. Pathogenic variants in dominant NDD‐associated genes have been detected in affected individuals yet inherited from phenotypically unaffected parents [45]. Additional population studies link such variants to mild developmental phenotypes in unselected cohorts [46]. Several of these genes encode other components of the epigenetic machinery, such as KMT2C, KMT2E, and MBD5. Interestingly, marked redundancy in function of several components exists, yet this is usually not sufficient to compensate for haploinsufficiency of a particular player. The majority of MDEMs is caused by heterozygous variants, where the affected individual is still expected to produce 50% of normal protein. It has therefore been hypothesized that these variants disrupt a delicate balance of histone marks and subsequent time‐ and tissue‐dependent gene expression [47]. This might also explain the reduced penetrance and variable expressivity observed in the KDM2B‐associated disorder, where some individuals have, potentially influenced by other genetic or environmental factors, not reached the threshold of sufficient disturbance of gene expression levels to develop symptoms.

Penetrance may be modulated by multiple factors, including (common) genetic variants in other genes, epigenetics, somatic mosaicism, environmental factors, lifestyle, and sex [48]. Interestingly, in our study, all but 1 variant were inherited from mildly or unaffected males to more severely affected daughters. A female predominance was present in the 12q24.31 microdeletions group (11 females vs. 3 males). These observations align with findings from a *KDM2B* mouse model demonstrating a correlation between phenotype severity and sex [49]. Interestingly, a significant male overrepresentation was reported in the SETD1B cohort [24] further supporting the need for investigation into potential sex‐specific effects in MDEMs. These findings underscore the importance of not discarding a variant based solely on inheritance and highlight the limitations of exome‐wide *de novo* filtering.

A recent study describes the association of an NDD and heterozygous variants in *KDM2A*, a close homolog of *KDM2B* [50]. Corresponding to *KDM2B*‐NDD, *KDM2A* pLOF, JmjC and CxxC variants are associated with a *KDM2A* episignature, while other variants were not [50]. Functional studies have shown *KDM2A* JmjC variants decrease protein stability [50, 51], including the residue homologous to *KDM2B* p.(Arg349Pro) [27, 51]. These findings for *KDM2A* suggest that JmjC variants primarily affect protein stability, acting like LOF variants.

Our findings support *KDM2B* as a strong contributor to the 12q24.31 microdeletion syndrome, together with other genes within the deletion, especially *HNF1A* and *SETD1B*. This suggests the 12q24.31 microdeletion syndrome is a contiguous gene syndrome, or genomic disorder, where the loss of multiple dosage sensitive genes contributes to phenotypic variability.

Epilepsy is a main feature of the monogenic *SETD1B*‐associated NDD [24]. Conversely, only five of 11 individuals with *SETD1B* included in their 12q24.31 microdeletion have epilepsy. Although these are relatively small numbers, the epilepsy phenotype does not appear fully penetrant in the 12q24.31 microdeletion syndrome. Interestingly, four of six individuals with a *SETD1B* deletion yet without epilepsy also have *RNF34* included in the deletion. *RNF34* knockdown has been shown to increase GABAergic innervation in cultured neurons [52], pointing towards a potential protective effect.

Defects in *HNF1A* are known to cause MODY3, characterized by reduced insulin secretion and subsequent hyperglycemia [53]. All five individuals with deletions including *HNF1A* had (a period of) obesity or diabetes and one individual presented with neonatal hypoglycemia due to hyperinsulinism. Additional cases are described where variants in *HNF1A* are implicated in congenital hyperinsulinism and neonatal hypoglycemia [54]. In murine models, *HNF4A* deficiency has shown temporally distinct effects: from promoting insulin secretion during fetal and neonatal periods to preventing insulin secretion and leading to beta‐cell loss in adulthood [54]. A similar temporal mechanism may underlie the clinical variability associated with *HNF1A* haploinsufficiency.

In summary, we analyzed the variant and phenotypic spectrum of the *KDM2B*‐associated NDD, emphasizing genotype–phenotype correlations and reduced penetrance. Our findings have implications for KDM2B variant classification and genetic counseling and stress the importance of further studies into the functional effects of KDM2B variants.

## Author Contributions

Conceptualization: R.O., A.S.E.O., T.‐C.H., B.P.C.K.; Data curation: R.O., A.S.E.O.; Formal analysis: A.S.E.O., T.‐C.H.; Funding acquisition: R.O.; Investigation: R.O., A.S.E.O., T.‐C.H., M.K., J.‐M.L; Methodology: R.O., A.S.E.O., T.‐C.H., B.P.C.K.; Resources: A.A., F.A., E.B., T.S.B., Y.C., S.C.‐B., V.C., D.A.C., E.C., N.E., M.F., N.M., V.M., S.G.M., M.N., C.P., N.R., M.S., N.S., J.S., P.V., S.W., F.Z., M.A.; Supervision: R.O., B.P.C.K.; Visualization: A.S.E.O., T.‐C.H.; Writing – original draft: A.S.E.O.; Writing – review and editing: R.O., A.S.E.O., B.P.C.K., A.A., E.B., T.S.B., D.A.C., E.C., N.E., V.M., M.N., C.P., N.R., M.S., S.W., M.A.

## Funding

This work was supported by the Wilhelmina Children's Hospital Research Fund [WKZ onderzoeksfonds 2020‐2021 to R.O.]. IRCCS “G. Gaslini” is a member of ERN‐Epicare. The Barakat lab thanks Stichting 12q (https://stichting12q.nl/) for supporting our work related to disorders on chromosome 12q. Part of this study was supported by NextGenerationEU—Italian Ministry of University and Research (MUR), National Recovery and Resilience Plan (PNRR)—funded research programs (to E.B. and M.V.): CN00000041—Project “National Center for Gene Therapy and Drug‐based on RNA Technology” (CUP J33C22001140001) and MNESYS (PE0000006)—“A Multiscale integrated approach to the study of the nervous system in health and disease” (DN. 1553 11.10.2022).

## Ethics Statement

This study was reviewed and approved by the medical ethical committee of the University Medical Centre Utrecht (TCBIO 21‐355, March 18, 2021). Informed consent for publication was obtained from all participants or their legal representatives. For all novel participants whose photographs have been used in this manuscript, explicit written informed consent for publication has been obtained.

## Conflicts of Interest

D.A.C. is an employee of and may own stock in GeneDx. The other authors declare no conflicts of interest.

## Supporting information


**Figure S1:** Expression of KDM2B long (LF) with CxxC deletion (△CxxC). (A, B, C) Western blot results of wild‐type (WT) and mutant His‐KDM2B‐LF△CxxC expression in HEK293T cells following transfection with WT or mutant His‐KDM2B expression plasmid. Cells were treated with increasing concentrations of the proteasome inhibitor MG132 (2.5, 5, 10 and 20 μM). Tubulin was used as a loading control. Untransfected samples were included as negative controls.
**Figure S2:** Facial images of individuals with a CxxC variant which is depicted below their image. Common facial features include long arched eyebrows, downslanted palpebral fissures, a bulbous nasal tip, a prominent cupid's bow, full cheeks, and asymmetric or dysplastic ears and prominent ear lobes.
(A–E) previously published in Van Jaarsveld et al. Genet Med. 2023 January; 25 (1): 49‐62. doi: 10.1016/j.gim.2022.09.006. Reprinted with permission from Elsevier. (F–G) previously published in van Oirsouw et al., Hum Mol Genet. 2025 August 16; 34 (16):1353–1367. doi: 10.1093/hmg/ddaf082. This is an open access article distributed under the terms of the Creative Commons CC BY license, which permits unrestricted use, distribution, and reproduction in any medium, provided the original work is properly cited.
**Table S1:** Clinical and genetic data of 13 previously undescribed individuals with KDM2B variants.
**Table S2:** All individuals and KDM2B variants included in this study (*n* = 68).
**Table S3:** KDM2B episignature results.
**Table S4:** ACMG classification of previously undescribed individuals and individuals for which novel data is now available.
**Table S5:** Clinical and genetic data of individuals with 12q24.31 microdeletions.
**Table S6:** Overview of haploinsufficient genes (loss‐of‐function observed/expected upper bound fraction (LOEUF) threshold of < 0.6) included in the 12q24.31 microdeletions.

## Data Availability

The data that support the findings of this study are available on request from the corresponding author. The data are not publicly available due to privacy or ethical restrictions.
