# Supplementary material for: Comprehensive Assessment of the KDM2B‐Associated Neurodevelopmental Disorder and the 12q24.31 Microdeletion Syndrome
Source: Clin Genet. 2026 Apr 9;110(2):150–64. doi: 10.1111/cge.70169 (PMC13327229; doi:10.1111/cge.70169)
Supplement: Supplementary file 1 — Figure S1: Expression of KDM2B long (LF) with CxxC deletion (△CxxC). (A, B, C) Western blot results of wild‐type (WT) and mutant His‐KDM2B‐LF△CxxC expression in HEK293T cells following transfection with WT or mutant His‐KDM2B expression plasmid. Cells were treated with increasing concentrations of the proteasome inhibitor MG132 (2.5, 5, 10 and 20 μM). Tubulin was used as a loading control. Untransfected samples were included as negative controls. Figure S2: Facial images of individuals with a CxxC variant which is depicted below their image. Common facial features include long arched eyebrows, downslanted palpebral fissures, a bulbous nasal tip, a prominent cupid's bow, full cheeks, and asymmetric or dysplastic ears and prominent ear lobes. (A–E) previously published in Van Jaarsveld et al. Genet Med. 2023 January; 25 (1): 49‐62. doi: 10.1016/j.gim.2022.09.006. Reprinted with permission from Elsevier. (F–G) previously published in van Oirsouw et al., Hum Mol Genet. 2025 August 16; 34 (16):1353–1367. doi: 10.1093/hmg/ddaf082. This is an open access article distributed under the terms of the Creative Commons CC BY license, which permits unrestricted use, distribution, and reproduction in any medium, provided the original work is properly cited. Table S1: Clinical and genetic data of 13 previously undescribed individuals with KDM2B variants. Table S2: All individuals and KDM2B variants included in this study (n = 68). Table S3: KDM2B episignature results. Table S4: ACMG classification of previously undescribed individuals and individuals for which novel data is now available. Table S5: Clinical and genetic data of individuals with 12q24.31 microdeletions. Table S6: Overview of haploinsufficient genes (loss‐of‐function observed/expected upper bound fraction (LOEUF) threshold of < 0.6) included in the 12q24.31 microdeletions. [file CGE-110-150-s001.zip › Supplements.docx]

# Supplementary information

## Legends to supplementary figures and tables

Figure S1: Expression of KDM2B long (LF) with CxxC deletion (ΔCxxC). (A, B, C) Western blot results of wild-type (WT) and mutant His-KDM2B-LFΔCxxC expression in HEK293T cells following transfection with WT or mutant His-KDM2B expression plasmid. Cells were treated with increasing concentrations of the proteasome inhibitor MG132 (2.5, 5, 10 and 20 μM). Tubulin was used as a loading control. Untransfected samples were included as negative controls.

Figure S2: Facial images of individuals with a CxxC variant which is depicted below their image. Common facial features include long arched eyebrows, downslanted palpebral fissures, a bulbous nasal tip, a prominent cupid’s bow, full cheeks, and asymmetric or dysplastic ears and prominent ear lobes.

A-E previously published in Van Jaarsveld et al. Genet Med. 2023 Jan;25(1):49-62. doi: 10.1016/j.gim.2022.09.006. Reprinted with permission from Elsevier. F-G previously published in van Oirsouw et al, Hum Mol Genet. 2025 Aug 16;34(16):1353-1367. doi: 10.1093/hmg/ddaf082. This is an open access article distributed under the terms of the Creative Commons CC BY license, which permits unrestricted use, distribution, and reproduction in any medium, provided the original work is properly cited.

Table S1: Clinical and genetic data of 13 previously undescribed individuals with *KDM2B* variants.

Table S2: All individuals and *KDM2B* variants included in this study (n=68).

Table S3: *KDM2B* episignature results.

Table S4: ACMG classification of previously undescribed individuals and individuals for which novel data is now available.

Table S5: Clinical and genetic data of individuals with 12q24.31 microdeletions.

Table S6: Overview of haploinsufficient genes (loss-of-function observed/expected upper bound fraction (LOEUF) threshold of <0.6) included in the 12q24.31 microdeletions.

## Supplementary methods

GestaltMatcher

Each image was encoded into a 12-dimensional Facial Phenotype Descriptor (FPD), and cosine distances between FPDs were calculated to quantify similarity in the Clinical Face Phenotype Space (CFPS), where smaller distances indicate greater similarity. Three main analyses were conducted: patient-level analysis, cohort-level intra group analysis, and cohort-level inter-group analysis ^1^. First, pairwise rank analysis assessed patient-level similarity by iteratively comparing each image against the other 19 study images and a gallery of 7,459 photographs from 449 syndromes in the GestaltMatcher Database (GMDB) ^2^. Second, intra-cohort similarity was measured by calculating mean pairwise distances within each group (CxxC, JmjC, microdeletion). We excluded comparisons involving the same individual and comparisons between related individuals from the same family to reduce non-independence and familial resemblance bias. We then compared these distances with two controls: a same-syndrome control and a random control. A ROC analysis of these controls yielded a threshold of c = 0.915, applied to determine whether groups showed a recognizable facial gestalt (when the distribution with 50% below threshold). Third, inter-cohort similarity compared groups by calculating mean pairwise distances between randomly sampled subcohorts. Two additional controls (same- vs. different-syndrome) provided context, and a separate ROC analysis defined a threshold of c = 0.896 for assessing shared similarity between groups (when the distribution with 50% below threshold).

DNA constructs and protein expression in HEK293T cells

In brief, the His-KDM2B-LF (ENSP00000366271 = NP_115979.3) and His-KDM2B-SF (XP_005254018.1) constructs were created with In-Fusion cloning (Takara 5X In-Fusion HD Enzyme Premix, Takara Bio), using GFP-FBXL10 (Addgene plasmid #126542) and a backbone including a EF1A promoter, P2A-EGFP-T2A-PuroR and AmpR (previously described ^3^). Agilent Quikchange II XL-Site directed mutagenesis kit (Agilent Technologies) was used to introduce patient variants in the wild-type KDM2B constructs. Transfection of wild-type and mutant His-tagged KDM2B-SF and KDM2B-LF was performed in HEK293T cells (n=3, Fig. 2, S1). HEK293T cells were grown in Dulbecco’s Modified Eagle’s Medium (DMEM) - high glucose (Sigma Aldrich) supplemented with 10% fetal bovine serum (Bodinco) and 1% penicillin-streptomycin (Gibco). Cells were incubated at 37°C in 5% CO2, 95% air in humidified cell culture incubators. HEK293T cells were seeded on day 1 in 12-well plates at 30-40% confluency. The next day, cells were transiently transfected using 1 µg plasmid DNA (KDM2B-SF/LF WT or variants) with 3 µl PEI (1 mg/ml) (Sigma Aldrich) in 100 µl Opti-MEM (Gibco). On day 3, cells were harvested and lysed with 100 µl Pierce RIPA buffer (Thermo Scientific, 89900), containing protease inhibitor (cOmplete Mini, EDTA-free protease inhibitor cocktail tablets, Roche, 11836170001). Samples were sonicated 3x30 seconds at 4°C at a low frequency with the BioRuptor Pico. SDS-PAGE was performed using Mini Gel Tank from Invitrogen Thermofisher, with a NuPage 4-12% Bis tris gel (1 mm x 15 well) (Invitrogen) for KDM2B-SF or a NuPage 3-8% Tris-Acetate gel (Invitrogen) for KDM2B-LF. Transfer was via a BIO-RAD Trans-Blot Turbo to a 0.2 μm nitrocellulose mini (BIO-RAD) at 25V, 1.3A for 10 minutes. Primary antibodies for GFP (Sigma SAB4301138, 1:5000), 6x-His-tag (Invitrogen MA1-21315, 1:2000), α-tubulin (Invitrogen, 1:2000) were used, with the secondary antibodies Goat anti-Rabbit IRDye® 800CW (LICOR 926-32211, 1:7500) and Goat anti-Mouse Alexa Fluor 680 (Invitrogen A21057, 1:7500). The blots were scanned with the Amersham Typhoon scanner.

## Case reports

Individual #1 is a 9-year-old girl with a *de novo* 0.828 Mb deletion of 22 protein- and non-protein-coding genes, including *KDM2B* (Figure 4A). She has moderate global DD and ID, with an IQ of 55-60 estimated at age 4 years. At age of 8y10m, she has a developmental age of 3.1 years, can speak in short sentences and was formally diagnosed with ASD and ADHD. At 6 years episodes of possible absence seizures were reported. A 24h EEG showed multifocal and generalized epileptiform activity but no clinical seizures. The first generalized seizure occurred at age 8 years and 9 months. Initially treated with levetiracetam but given the side effects switched to valproic acid. Brain imaging was normal. Facial features comprised low anterior hairline, mild hypotelorism, epicanthal folds, short philtrum and thick lip vermilion.

Individuals #2 is a 3-year-old girl with a *de novo* 0.356 Mb deletion of 8 protein- and non-protein-coding genes, including *KDM2B*. She has ID, mild motor delay and moderate speech delay. She started walking at 2 years, with unsteady gait, tiptoeing and a low tone in lower limbs, and is currently non-verbal. Autistic features were noted, including social communication delay, however no formal diagnosis was made. She has difficulty swallowing lumpy foods, dribbles and suffers from constipation. An ultrasound of the kidney and urinary tract showed a prominent column of Bertin bilaterally, but not a definite duplex system, and a mildly prominent renal pelvis bilaterally (5 mm AP diameter) which does not extend into the calyces. She has esotropia on her right eye and a refractive error. She has obstructive sleep apnea and has had tonsillectomy and adenoidectomy. Cardiac echo was normal. Physical examination noted a flat occiput, fading nevus simplex on back of head, arched eyebrows, synophrys, round face with full cheeks, anteverted nares, macroglossia, cutis marmorata, broad toes and sandal gap. Facial photograph is shown in Fig. 1B.

Individual #3 is a 12-year-old boy with a *de novo* in-frame *KDM2B* deletion p.(Asn579_Ala653del), spanning exon 13 that encompasses the CxxC domain. He has mild to moderate ID and global DD with marked speech delay. Behavioral assessments revealed ASD, autism-related sensory issues, anxiety and hair pulling. He had a history of a very small PDA and a small atrial septal defect, which spontaneously closed. He was diagnosed with cryptorchidism, bilateral iridocorneal adhesions and recurrent epistaxis. He received grommets for his recurrent otitis media. Facial features comprised downslanted palpebral fissures, midface retrusion and ears with large lobules. Additionally, pes planus was noted. Brain imaging was normal.

Individual #4 is a 27-year-old female with a *KDM2B* variant of unknown inheritance: c.1936dup; p.(Cys646Leufs*37). She was diagnosed with depression, borderline personality disorder, complex PTSD and ADHD. Autistic features were also noted but not formally diagnosed. She uses a walker, without clear reason, and reports chronic pain in her back and legs. No developmental delays were noted. She has a history of surgery for craniosynostosis, VSD that closed spontaneously, asthma, astigmatism, GERD, multiple renal cysts and a splenic cyst. Physical features include a small head circumference (-1.94 SD), facial asymmetry related to left coronal craniosynostosis, broad forehead, deeply set eyes, hypertelorism, narrow concave nasal ridge, bilateral sandal gap, proximally placed 5th toes and congenital radioulnar fusion (left).

Individual #5 is a 22-year-old female with *KDM2B* variant p.(Val316Ile), inherited from her mildly affected father ^4^. She was born after 33 weeks and 5 days with a congenital deformity of the left leg, following a twin pregnancy. She was diagnosed with unilateral bowing of tibia (45 degrees), corrected with brace therapy (Fig 1C).

Her twin brother and older sister also carry the identical *KDM2B* variant, and both have ID and the brother has epilepsy. She had a normal development. Dyslexia was suspected, but never formally diagnosed. She has asthma and pollen allergy.

Individual #6 is an 8-year-old boy with a *de novo KDM2B* missense variant p.(His253Arg). He was born at term with birth parameters below average (SD ± -1.5). During development, the growth failure became more severe (SD < -3). He had feeding difficulties requiring percutaneous endoscopic gastrostomy tube placement. He presented with severe global DD, achieving head control at 3.5 years, sitting at 4 years, unassisted walking at 6 years and first words at 7 years. He has severe ID and psychomotor delay. Brain imaging revealed septo-optic dysplasia, hypoplasia of the optic nerves and a dysmorphic corpus callosum. He was diagnosed with adrenal insufficiency, nystagmus, chorioretinal dystrophy and strabismus. Facial features comprised microcephaly, sparse eyebrows, blepharophimosis, long eyes with narrow palpebral fissures, depressed nasal tip, low-set ears, thin lip vermillion and a smooth philtrum. Additional physical features include a dry skin, syndactyly of the 2nd-3rd toes, clubfoot (right), clinodactyly of the 4th-5th toes and a single palmar crease.

12q24.31 microdeletion syndrome

The core phenotype of the 12q24.31 microdeletion syndrome includes DD, ID, behavioral problems and various dysmorphisms (Tables 1, 2, S5, Supplemental text). Most individuals in this cohort presented with global DD (14/15), including marked speech delay (12/14), motor delay (8/11), and ID (12/14). Behavioral problems were reported in 13 out of 14 individuals, with high rates of ASD or autistic features (10/14), anxiety (6/14), and ADHD (2/14). Epilepsy was observed in half of the individuals (7/14). Growth abnormalities were not consistent (Fig. 4B), aside from a tendency to a small head circumference (median z-score -1.28; 3/12 microcephaly). Congenital anomalies were present in several individuals, including cardiac (3/13), ophthalmological (3/12), and urogenital (3/12) abnormalities. Other recurrent phenotypic features included macroglossia (7/13), obesity (4/9), diabetes (4/15), and neonatal hypoglycemia (2/14). The presence of macroglossia and/or neonatal hypoglycemia led to an initial clinical suspicion of Beckwith–Wiedemann syndrome in 3 individuals.

## References

1. Mak CCY, Klinkhammer H, Choufani S, et al. Artificial intelligence-driven genotype–epigenotype–phenotype approaches to resolve challenges in syndrome diagnostics. *EBioMedicine*. 2025;115. doi:10.1016/j.ebiom.2025.105677

2. Lesmann H, Hustinx A, Moosa S, et al. GestaltMatcher Database - A global reference for facial phenotypic variability in rare human diseases. *medRxiv*. 2024;25:2023.06.06.23290887. doi:10.1101/2023.06.06.23290887

3. Dirkx N, Weuring WJ, De Vriendt E, et al. Increased prime edit rates in KCNQ2 and SCN1A via single nicking all-in-one plasmids. *BMC Biol*. 2023;21(1). doi:10.1186/S12915-023-01646-7

4. van Jaarsveld RH, Reilly J, Cornips MC, et al. Delineation of a KDM2B-related neurodevelopmental disorder and its associated DNA methylation signature. *Genet Med*. 2023;25(1):49-62. doi:10.1016/J.GIM.2022.09.006
